# Uneven Terrain Recognition Using Neuromorphic Haptic Feedback

**DOI:** 10.3390/s23094521

**Published:** 2023-05-06

**Authors:** Sahana Prasanna, Jessica D’Abbraccio, Mariangela Filosa, Davide Ferraro, Ilaria Cesini, Giacomo Spigler, Andrea Aliperta, Filippo Dell’Agnello, Angelo Davalli, Emanuele Gruppioni, Simona Crea, Nicola Vitiello, Alberto Mazzoni, Calogero Maria Oddo

**Affiliations:** 1 The BioRobotics Institute, Sant’Anna School of Advanced Studies, 56127 Pisa, Italy; 2 Department of Excellence in Robotics & AI, Sant’Anna School of Advanced Studies, 56127 Pisa, Italy; 3 Interdisciplinary Research Center Health Science, Sant’Anna School of Advanced Studies, 56127 Pisa, Italy; 4 Centro Protesi INAIL (Italian National Institute for Insurance against Accidents at Work), 40054 Budrio, Italy; 5 IRCCS Fondazione Don Carlo Gnocchi, 50143 Florence, Italy

**Keywords:** neuromorphic haptic feedback, FPGA neuron model, Izhikevich, terrain recognition, PSTH-based classification, tactile augmentation, wearable assistive robotics, lower-limb impairments

## Abstract

Recent years have witnessed relevant advancements in the quality of life of persons with lower limb amputations thanks to the technological developments in prosthetics. However, prostheses that provide information about the foot–ground interaction, and in particular about terrain irregularities, are still missing on the market. The lack of tactile feedback from the foot sole might lead subjects to step on uneven terrains, causing an increase in the risk of falling. To address this issue, a biomimetic vibrotactile feedback system that conveys information about gait and terrain features sensed by a dedicated insole has been assessed with intact subjects. After having shortly experienced both even and uneven terrains, the recruited subjects discriminated them with an accuracy of 87.5%, solely relying on the replay of the vibrotactile feedback. With the objective of exploring the human decoding mechanism of the feedback startegy, a KNN classifier was trained to recognize the uneven terrains. The outcome suggested that the subjects achieved such performance with a temporal dynamics of 45 ms. This work is a leap forward to assist lower-limb amputees to appreciate the floor conditions while walking, adapt their gait and promote a more confident use of their artificial limb.

## 1. Introduction

In intact subjects, the afferent information about the foot–ground interactions is continuously gathered by the plantar mechanoreceptors and conveyed to the Central Nervous System (CNS), thus aiding balance regulation and posture maintenance during gait and stance [1,2]. The absence of such sensorimotor information presents a major threat to the quality of life and safety of lower-limb amputees, since it compromises their gait functions, balance performance and locomotion confidence [3].

Although the recent advancements in the development of prosthetic devices are providing promising evidence for restoring natural locomotion behaviors [4,5,6], the lack of plantar information increases both cognitive load and energy consumption [7,8]. Sensory remapping and augmentation approaches can compensate for this loss by partially recovering the afferent signals by means of artificial invasive [9,10] and non-invasive [11,12] feedback systems. Non-invasive systems are inherently safer and more user-friendly, although they do not evoke refined sensations [3]. Among them, the devices that deliver visual cues are the most common [13]. Notwhitstanding that, when a person loses her/his own limb, the undamaged sensory channels are usually overburdened, since she/he relies more on them to enhance her/his own self-confidence in using the prosthesis. In addition, stimuli that elicit natural-like sensations are more desirable and faster to learn [14,15]. In this scenario, non-invasive haptic systems fulfil these basic requirements, conveying high-quality spatiotemporal and intuitive information [16]. Vibrotactile displays are usually preferred to other touch-perception-based technologies [17]. In fact, they can be mapped into the sensorymotor scheme of the user in a straightforward manner and provide rich sensory contents through the tuning of multiple parameters, such as the amplitude, the frequency and the duration of the activations. In addition, their small form factor and low cost make them appealing for many commercial and daily-life applications [17,18].

So far, most of the relevant studies have been devoted to promoting postural and balance control [19,20,21,22], improving gait symmetry [12,23,24,25] and enhancing walking performance [26]. Less evidence has been reported about the perception of the floor conditions, although the identification of the properties of a terrain to walk on is crucial for the dynamic stability of a person with amputation [27]. As a matter of fact, the uneveness of the ground is one of the most significant environmental factors affecting the risk of falls [28,29,30]. Whilst several research works on terrain recognition to reliably control the prostethic device are reported in the literature [4,31,32,33,34,35], fewer examples deal with sensory-feedback-based approaches. In [15], the authors demonstrated that the combination of a virtual reality environment and vibrotactile sensory feedback effectively gave paraplegic patients the sensation of walking on different surfaces. Another study showed that a group of subjects with unilateral amputations significantly improved the recognition of under-the-foot objects when receiving vibratory cues [36]. However, to the best of our knowledge, overground walking supported by sensory feedback to adjust the gait pattern in reponse to path obstacles has not been tested yet. The subject’s awareness of the surroundings can be enhanced through the artificial restoration of the plantar perception to elict prompt reactions to different terrain conditions during autonomous exploration. This might foster amputees’ closer-to-naturalistic locomotion, entailing less effort in the interaction with varied ground textures [30].

In this perspective, the present study primarily attemps to evaluate whether a neuromorphic vibrotactile feedback strategy can assist users in the identification of even and uneven (i.e., rocky) terrains. For this purpose, a waist haptic belt, featuring three vibrotactile units, was used to deliver stimuli synchronized with the foot–ground interactions captured by a sensorized insole placed in the shoe. To achieve an intuitive perception of the terrain structure, a novel neuromorphic feedback strategy that complies with a biologically plausible neuronal model is herein proposed. Specifically, the behavior of the foot mechanoreceptors was mimicked through the spatiotemporal encoding of the plantar tactile information into vibrations (i.e., spike trains). This biomimetic approach previously succeeded in invasive neural feedback to enable upper-limb amputees to intuitively perceive object textures [37,38,39]. According to the principle of contingency-mimetics, non-invasive sensory augmentation strategies that exploit sensorimotor contingencies can achieve fast and long-lasting perceptual effects [40,41,42]. Starting from this assumption, the natural-like neuromorphic coding scheme of the vibrotactile feedback was provided to able-bodied subjects while walking over different terrains. Then, the integration of the vibratory cues into the subjects’ perception of the floor condition was validated through a playback phase, where the previously experienced feedback patterns were delivered to the participants. Finally, this study aims also at elucidating the feedback features that are involved in the human mechanism of distinguishing between even and uneven terrains and for recognizing them among tiles, grass and stones. Specifically, the explored vibration features encoded the relative changes in the foot–ground contact during the gait cycle and the amount of associated cues carried by individual or combinations of tactile units.

## 2. Materials and Methods

**Figure 1:**
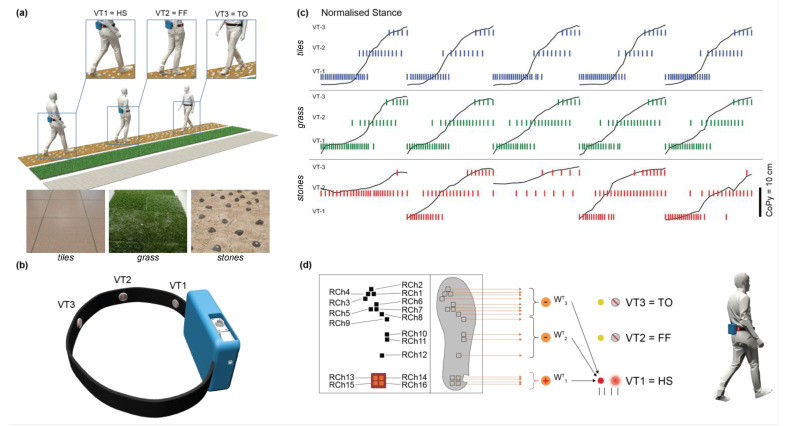
Experimental setup overview. (**a**) Familiarization phase: Exploration of the three terrains, i.e., tiles, grass and stones (terrain configurations in the pictures). The detection of gait events by the insole activates the corresponding VTs as follows: VT1 is triggered at the heel–strike (HS); VT2 is triggered at the foot-flat (FF); and VT3 is triggered at the toe-off (TO). (**b**) The wearable augmenting haptic belt embedding the VTs placed along the waist from the spine (VT1) to the navel (VT3). (**c**) Neuromorphic vibrotactile feedback: examples of real-time spike trains delivered by each VT unit according to the detected stance phase while walking along even (grass and tiles) and uneven (stones) floors. (**d**) Neuromorphic vibrotactile feedback computation: example of the activation of VT1 relying on the foot pressure sensors embedded in the insole and the relevant neuromorphic computation.

A wearable augmenting haptic belt was designed and developed to provide neuromorphic vibrotactile feedback about the foot–ground interaction during walking on different types of terrains (Figure 1a). The feedback consisted of temporal patterns of vibrations delivered on the waistline by three actuators, namely the VTs. To assess whether the subjects could leverage this information to perceive their navigation on diverse terrains, psychophysical indoor experiments were carried out with four male able-bodied volunteers. The participants (age: 29 ± 4) were recruited among the personnel from Sant’Anna School of Advanced Studies. The inclusion criteria concerned the foot size, in the range of 40–43 (EU), because of the form factor of the sensing insole, and the absence of any sensory and motor impairments. The psychophysical experiment consisted of a familiarization and a playback phase, requiring locomotion and recognition tasks, respectively.

### 2.1. Experimental Setup

The wearable haptic feedback system consisted of three main components: a sensing shoe insole, a tactile display and a haptic control unit [43,44].

The sixteen optoelectronics sensors embedded in the insole read the applied pressure distribution resulting from the foot–ground interactions. The voltage readings were converted into force signals through a characteristic curve, as defined in [45]. The sensor configuration over the insole surface achieved the reliable detection of the gait events from the ground reaction force profile, as detailed in previous studies [45,46]. A dedicated electronic board placed on the shoe gathered these signals to wirelessly send them to a central computing unit, namely the Vibro Board (NI SOM 9651). Here, at the low level, a FPGA (Field-Programmable Gate Array) processed the plantar readouts to code them into neuronal spike trains that activated the haptic interface. This consisted of an adjustable textile belt embedding three Eccentric Rotating Mass (ERM) motors (Precision Microdrives™, London, United Kingdom) encapsulated in a silicone (PDMS) cover to enhance the comfort of the user. Henceforth, we refer to them as VT (vibrotactile) units. The belt was placed around the waist and the VTs were attached to its inner side by Velcro straps. Since the feedback is intended to provide the amputees with sensory information from the impaired limb, the VTs were just placed on one side of the waist, going from the spine (VT1) through the hip (VT2) to the navel (VT3). The distance between neighbor VTs was 9 cm, as in [47], according to the waist perceptual spatial resolution found in [48] (Figure 1b).

Three floor textures were settled indoors to reproduce realistic terrains. Tiles were replicated by the laboratory floor, grass by artificial plastic grass (IKEA, product: 503.131.31) and stones by climbing holds (ChatNoirClimb). The latter had an averaged dimension of 5 cm × 5 cm × 2.5 cm and they were randomly mounted on 12 particle boards (62.5 cm × 83 cm × 0.9 cm each) that were alternated per density level (23.4 holds/m^2^ and 39 holds/m^2^). Stones represented the uneven surface, whereas tiles and grass were comparatively uniform but different in their stiffness. Each terrain pathway was 7.5 m long to elicit the sensation of walking along (Figure 1a).

### 2.2. Feedback Strategy and Spiking Neuronal Network Architecture

In order to biomimetically encode the foot–ground interaction information onto the VT activations, a neuromorphic stimulation strategy was implemented (Figure 1c,d). The design of the proposed neuronal network took into consideration the mapping of both the spatial placement and the forces exerted by the ipsilateral foot, onto the waist through the spatially distributed VTs. Hence, at each discrete phase of the gait cycle (heel-strike, foot-flat and toe-off) detected by the plantar sensors, the spatially corresponding VT unit (VT1, VT2 and VT3, respectively) was activated (Figure 1d).

The raw data from the sensing insole were encoded into real-time spike trains generated by three Izhikevich artificial neurons [49], each one related to a discrete gait phase and hence to a VT unit. The neurons had a regular spiking dynamics updated at 16 kHz (model designed in LabVIEW-FPGA, National Instruments Corp, Austin, TX, USA), as in [44,49]. In more detail, the sensor data, $x^{n}$, were weighted to estimate the input, $I_{VTi}^{n}$, to each artificial neuron (Equation (1)). When the input triggered a spike in the neuron, the spike activated the corresponding VT for 15 ms, ignoring any other spike within this time window. The gains were experimentally tuned for each neuron to guarantee the feedback perception and avoid users’ discomfort. The resulting values, $g_{VTi}$, were 72.17 ms^−1^, 32.08 ms^−1^ and 38.42 ms^−1^ for VT1, VT2 and VT3, respectively, and they were set on a dedicated LabVIEW-RT GUI. The afferent receptive field weights, whose sign allowed for the appreciation of the stance phases, were defined as 𝑤^𝑇^_𝑉𝑇1_ = [−0.17, −0.24, 1.00], 𝑤^𝑇^_𝑉𝑇2_ = [0, 0.98, 0] and 𝑤^𝑇^_𝑉𝑇3_ = [0.32, −0.08, 0]. These weights have been chosen and experimentally tuned from a pool of values generated through a Particle Swarm Optimization (PSO) approach [50]. The terrain recognition accuracy of a k-Nearest Neighbors (KNN, K = 1) classifier based on the Victor–Purpura distance [51] between spike trains was considered as the fitness function to be optimized by the PSO procedure.

(1) $$I_{VTi}^{n}=g_{VTi}max\left(w_{VTi}^{T}x^{n},0\right)$$

$$I_{VTi}^{n}=input\;current\;to\;the\;neuron\;associated\;with\;the\;i^{th}VT;\;g_{VTi}=gain;\;w_{VTi}^{T}=weight;\;x^{n}=raw\;data\;from\;n=16\;sensors.$$

The three Izhikevich neurons (Equations (2) and (3)) were emulated on a Xilinx FPGA board that updated the membrane potential in accordance with the input, $I_{VTi}^{n}$, as follows:

(2) $$\frac{dv}{dt}=Av^{2}+Bv+C-u+{I_{VTi}^{n}}_{}$$

(3) $$\frac{du}{dt}=a\left(bv-u\right)$$

$$Whenv\geq Vth;then\left\{\begin{array}{c} v\leftarrow c\\ u\leftarrow u+d \end{array}\right.$$

where *v* is the membrane potential and *u* is the membrane recovery variable of the neuron. The constants for the model were *A* = 0.04 ms^−1^ mV^−1^; *B* = 5 ms^−1^; *C* = 140 mVms^−1^; *a* = 0.02 ms^−1^; *b* = 0.2 ms^−1^; *c* = −65 mV; *d* = 8 mV; *Vth* = 30 mV; *dt* = 0.0625 ms, with reference to [44,49].

**Figure 2:**
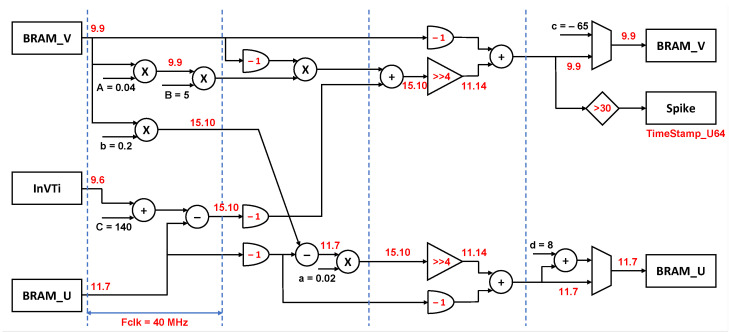
The customized fixed-point pipelined architecture designed for the Izhikevich neuron. The InVTi, BRAM_V and BRAM_U store the values of the input, $I_{VTi}^{n}$, *v* and *u*, respectively. The red numbers represent the fixed-point representation at every computational unit and the dotted lines denote the computational cycles.

The computational neuronal core included the piece-wise solution to the ODE in 5 parallel and pipelined stages (dotted vertical lines in Figure 2). Each stage was executed in one cycle of the FPGA, i.e., 25 ns. The fixed-point-based architecture (red values in Figure 2) facilitated the accurate and timely completion of data computation at each stage with an optimized usage of power and hardware resources. This approach can enable the implementation of 2500 other neurons on the same core of the chosen hardware, to implement even more refined neuromorphic strategies.

### 2.3. Experimental Protocol and Psychophysics

The experimental sessions of the terrain recognition task included two phases, i.e., familiarization and playback, lasting 100 min overall. During the familiarization, after having worn the augmenting haptic feedback device, the participants were asked to select the preferred parameters of the stimuli, i.e., the intensity and the duration of the vibrations. Then, every subject chose her/his own speed to walk at throughout the experiment, by adjusting her/his pace over the uneven terrain. The walking speed was then kept constant to not affect further recognition of the terrains. The familiarization included four blocks of training trials, each one consisting of a sequence of all the possible combinations of consecutive terrains to walk on, summing up to 12. The 48 total trials aimed at imparting the conceptual categorization of the perceived floor textures. The length of the paths allowed the subjects to perform five full stances on each terrain before starting the new trial, either on the same or a different terrain.

During the playback phase, instead, each subject was exposed to the vibratory stimulations generated and recorded during the 48 familiarization trials. The activations were pseudo-randomly delivered to the waist of the subject, who was in a comfortable stationary position. At each pattern, the subject was asked to identify the terrain type and her/his responses were collected by the experimenter through a dedicated LabVIEW GUI.

### 2.4. Psychophysical Data Analysis and Data Pre-Processing

The analysis aimed at investigating the subjects’ ability to distinguish even from uneven terrains and to recognize the three terrains as well. The responses of the subjects were analyzed to estimate the 95% confidence intervals (CIs) with the exact Clopper–Pearson method, and they were compared with the chance level (50% for unevenness recognition and 33% for terrain identification) to assess the performance significance. The differences in the walking behavior determined by the terrains were also assessed through a One-Way ANOVA on the duration of the subjects’ stances. In case of significant differences, post hoc comparisons were considered, and the relevant correction (Bonferroni’s) was applied. The significance level was α = 0.05. The analysis was performed in MATLAB (MathWorks, Inc., Natick, MA, USA).

### 2.5. Algorithm Decoding

**Figure 3:**
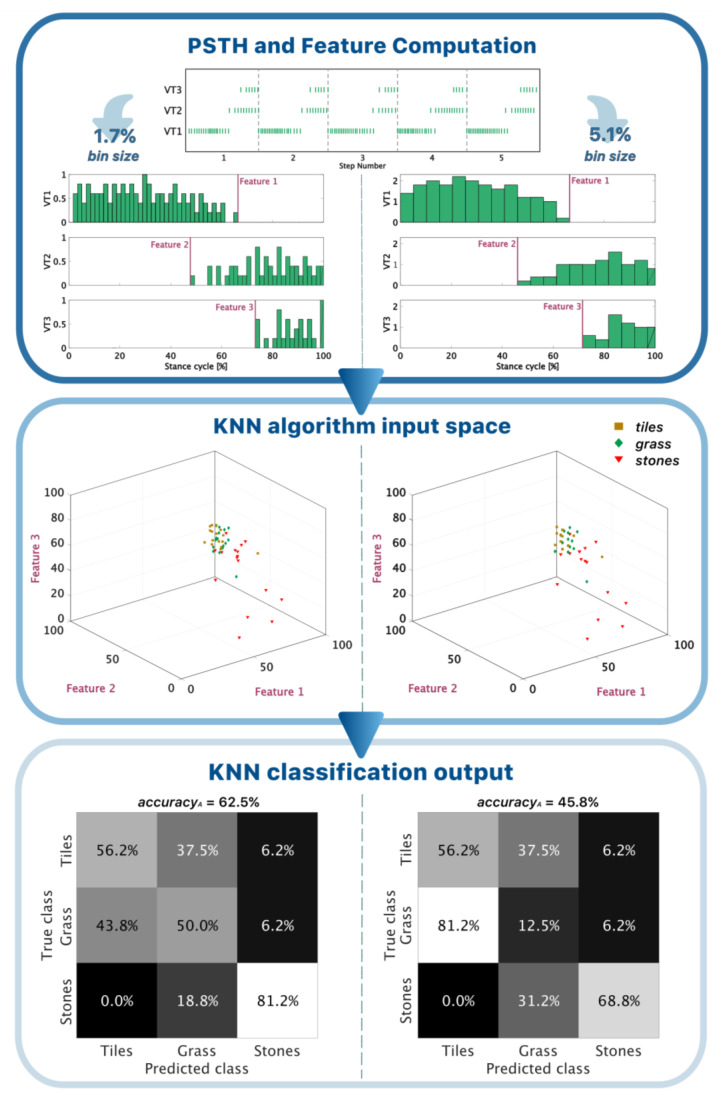
Workflow of the algorithm decoding. First box: the spike trains of each trial (i.e., 5 stances over a terrain) are pre-processed into the PSTHs for every bin size, ranging from 0% to 50% of the stance cycle. Two examples, for 1.7% (left column) and 5.1% (right column) bin sizes, are reported. The last activation time of VT1 (Feature 1) and the activation times of VT2 and VT3 (Feature 2 and Feature 3, respectively) are extracted and used as input features for the KNN algorithm. The second box represents the KNN input feature space for the two bin sizes. The last box shows the confusion matrix of the terrain classification task that the KNN algorithm outputs at each bin size.

The Peri-Stimulus Time Histograms (PSTH) of the spike trains related to each stance were extrapolated to decode the spatiotemporal information associated with the recognition of the terrains. Each trial was limited to five stances, whose spike times were normalized with respect to the total duration of each stance. The spikes (inter-spike interval = 41.4 ms ± 18.6 ms) were grouped by different bin sizes, which varied from 0% to 50% of the normalized stance cycle with a step of 1.7%. The step width was chosen according to the minimum inter-spike interval achievable (15 ms, i.e., the activation time of the VT at each single spike) and considering an averaged stance time of about 900 ms. Three features were then extracted for each bin size and fed to a machine learning algorithm to ultimately investigate the human mechanism of decoding of the vibrotactile feedback. Therefore, the deactivation (time of the last spike bin) of VT1 and the activations (time of the first spike bin) of VT2 and VT3 were chosen as they were considered, by visual inspection of the spike trains, to capture the differential walking behaviors across the terrains. The 3D feature vectors were then used to train population-wise (i.e., all the subjects included) and subject-wise (i.e., single subject) KNN classifiers (K = 5) to solve the terrain recognition task. In the case of subject-wise training, the model was cross-validated with a leave-one-out approach. The algorithm decoding workflow, so far, is represented in Figure 3.

For both the population- and subject-wise models, the accuracy of both the algorithm (accuracy_A_) and the human subjects (accuracy_H_) and the candidacy, that is, the overlap between the algorithm classifications and the subjects’ responses, were calculated as follows:

(4) $${accuracy}_{X}=\frac{\sum {TP}_{X}}{Number\;of\;trials}\times 100$$

(5) $$candidacy=\frac{\sum {TP}_{C}}{Number\;of\;trials}\times 100$$

where *TP_X_* and *TP_C_* were defined as

(6) $${TP}_{X}=\left\{\begin{array}{c} 1;if\;R_{X}==R_{T}\\ 0;\;ifR_{X}!=R_{T} \end{array}\right.$$

(7) $${TP}_{C}=\left\{\begin{array}{c} 1;\;ifR_{A}==R_{H}\\ 0;\;ifR_{A}!=R_{H} \end{array}\right.$$

Here, *R_A_* is the algorithm classification outputs, *R_T_* is the actual terrain and *R_H_* is the human subjects’ psychophysical responses. The *X* subscript in Equations (4) and (6) can stand for *A* (algorithm) or *H* (human), accordingly. The candidacy was calculated for every bin size and its maximum value determined the temporal scale, i.e., the corresponding bin size, needed for the recognition of the unevenness/terrains. Hence, the population candidate temporal resolution (CTRp) and the customized subject-wise candidate temporal resolution (CTRc) were extrapolated. The customized temporal transitions (CTTc) were then determined as the product of CTRc and the corresponding subject’s averaged stance length.

Finally, to investigate the impact of the VT activations on human terrain classification, the algorithm outputs were analyzed considering all the possible input combinations of VTs. For this purpose, the candidacy and the related CIs were computed for each VT group.

## 3. Results

### 3.1. Haptic Feedback for Discrimination of Uneven Terrains

During the familiarization, the participants were trained for the further task of unevenness/terrain recognition. They completed between four and seven stances, with a median value of five, taking 8.24 (5.4; 13) s (median, (min; max)) to accomplish each trial. Individual stances lasted (mean ± standard deviation) (780 ± 50) ms on tiles, (810 ± 80) ms on grass and (860 ± 180) ms on stones. The ANOVA on the stance durations returned significant differences (F (2137) = 6.23; *p* = 0.0026). The post hoc comparisons revealed that those differences concerned tiles and stones (*p* << 0.017, with the relevant correction of α) (Supplementary Materials Figure S1).

**Figure 4:**
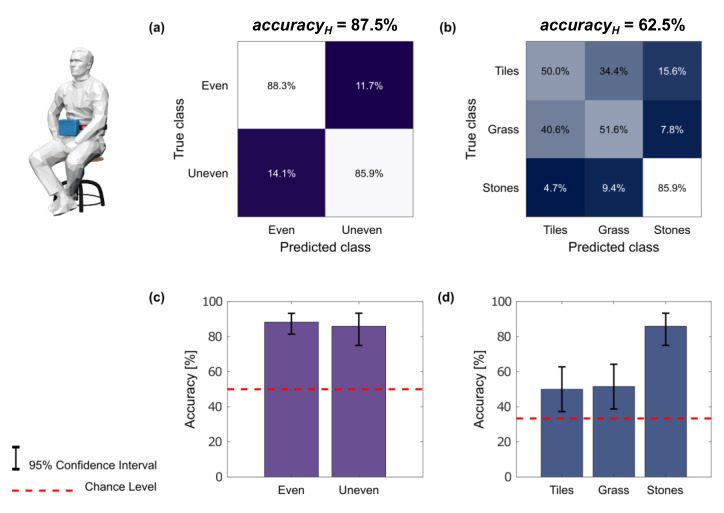
Terrain recognition and identification during playback: (**a**) confusion matrix of the subjects’ uneven terrain recognition; (**b**) confusion matrix of the subjects’ terrain identification; (**c**,**d**) accuracy_H_ and Clopper–Pearson exact intervals (error bars) for even/uneven terrain recognition and for each terrain type identification, respectively.

During the playback phase, the subjects distinguished between the even and the uneven terrains with an accuracy_H_ of 87.5% ± 11.7% (mean ± standard deviation) and they recognized the three terrains with an accuracy_H_ of 62.5% ± 15.7% (Figure 4). These outcomes were significantly better than the chance level for both the tasks (*p* < 0.05), as shown in Figure 4c,d. More details about the terrain recognition task in terms of the subject-wise confusion matrices are reported in Supplementary Materials Figure S2.

### 3.2. Decoding of the Neuromorphic Haptic Feedback for the Population-Wise Terrain Recognition

**Figure 5:**
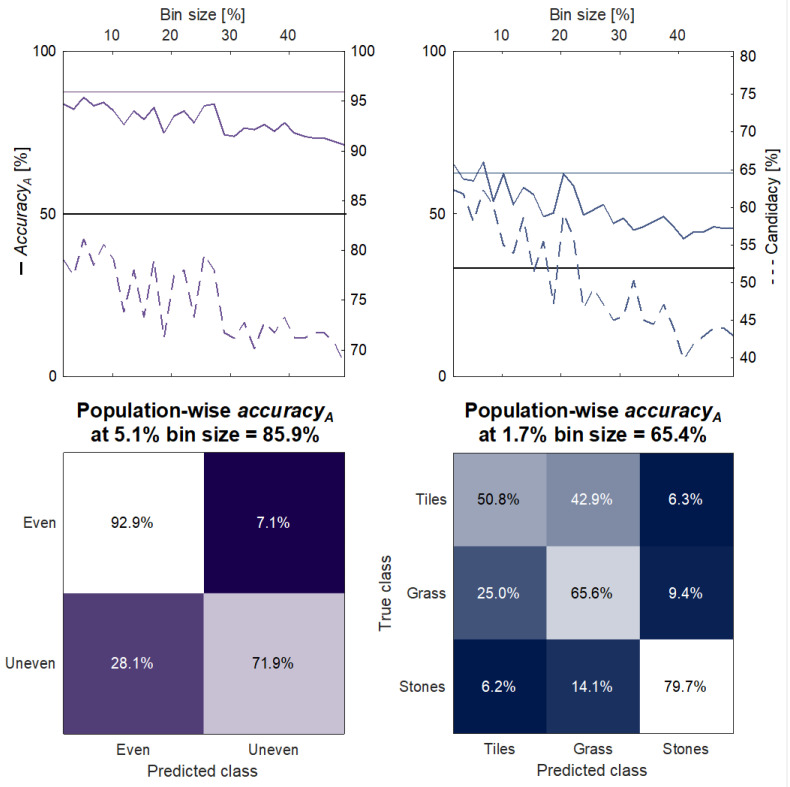
Population-wise algorithm decoding performance. Unevenness recognition performance (**left**) and three-terrain identification performance (**right**). Top: accuracy_A_ as a function of the bin interval measured as percentage of the stance duration (solid line); it is compared with subjects’ accuracy (shaded CI), with the chance level (flat solid line) and with the candidacy (right *y*-axis, dotted line). Bottom: confusion matrices of the algorithm classification output at the bin size corresponding to the maximum candidacy.

The population-wise KNN classification accuracy_A_ decreased with the increase in the bin size, i.e., the reduction in the temporal scale (R^2^ = 0.9260 and *p* < 0.05), when identifying the terrain. The confidence interval of the subjects’ psychophysical performance and the population-wise algorithm classification accuracy_A_ were found to overlap up to a bin size of 20–25% of the stance duration—for both the unevenness recognition and the terrain identification (Figure 5). The CTRp for the unevenness recognition was found to be 5.1% of the average duration of the stance, which corresponded to a 45 ms temporal dynamics. At this temporal scale, the algorithm candidacy was 83.8%, and its accuracy_A_ was 88.5% (Figure 5). Concerning the CTRp for the terrain identification, instead, it was found to be 1.7% of the stance duration, i.e., 15 ms, with an accuracy_A_ of 62.8% and a candidacy of 64.9%.

### 3.3. Estimation of the Human Decoding Temporal Dynamics through the Subject-Wise Algorithm Performance

**Figure 6:**
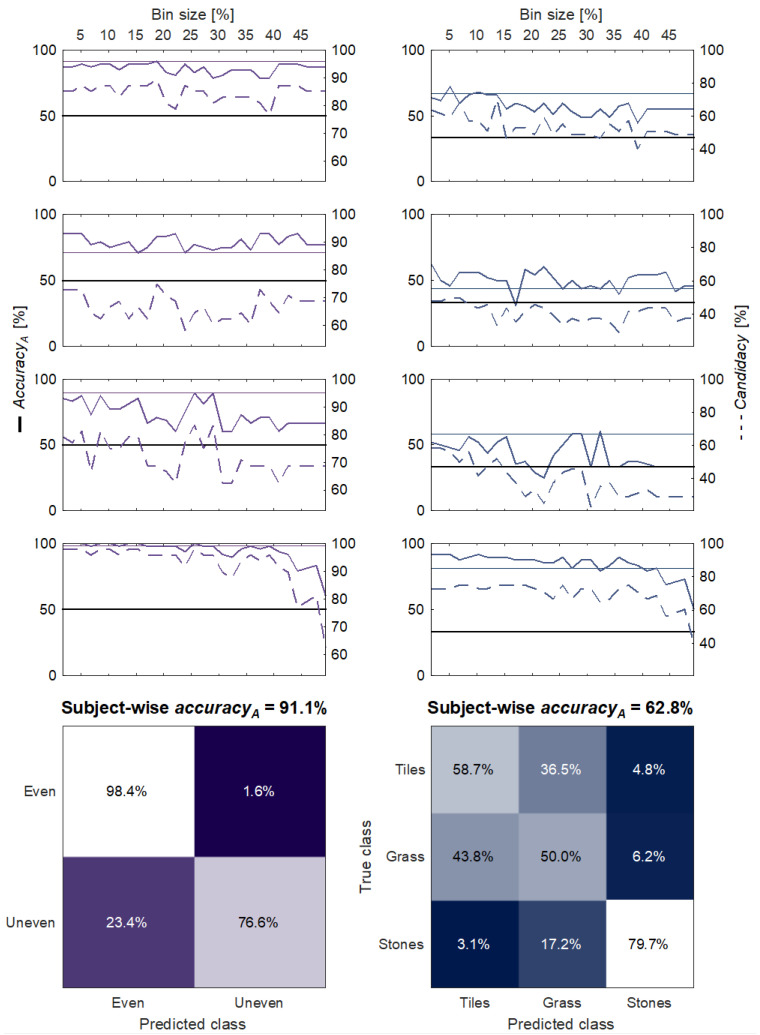
Subject-wise decoding performance. Unevenness recognition (**left**) and three-terrain identification performance (**right**). Top: accuracy_A_ (solid line) as a function of the bin size measured as percentage of the stance duration; it is compared with the subjects’ accuracy (shaded CI), with the chance level (flat solid line, 50% for the unevenness recognition and 33% for the terrain identification) and with the candidacy (right *y*-axis, dotted line). Bottom: averaged confusion matrices and accuracy_A_ of the algorithm classification output at the bin size corresponding to the maximum candidacy for each individual subject.

To investigate the human decoding temporal scale of the haptic feedback, the subject-wise analysis was carried out. Figure 6 shows the accuracy and the candidacy as a function of the bin size. The CTTc for the unevenness recognition was found to be 208 ms (CTRc = 25.5%), 14 ms (CTRc = 1.7%), 49 ms (CTRc = 5.1%) and 16 ms (CTRc = 1.7%) for subjects 1 to 4, respectively. In the case of terrain discrimination, the CTTc resulted to be 55 ms (CTRc = 6.8%), 14 ms (CTRc = 1.7%), 16 ms (CTRc = 1.7%) and 16 ms (CTRc = 1.7%). The across-subject averaged maximum candidacy and accuracy_A_ for the unevenness recognition were 86.4% ± 9.7% and 91.1% ± 6.9%, respectively. In the case of the terrain recognition task, they were 63.4% ± 11.3% (candidacy) and 62.8% ± 18.5% (accuracy_A_). Further details are reported in Table 1 and Table 2.

### 3.4. Effect of the Different Combinations of VTs

**Figure 7:**
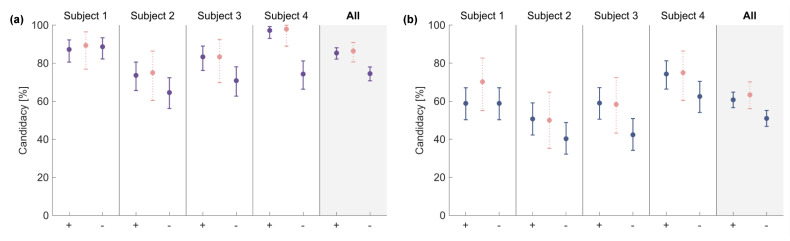
Effect of VT2 on algorithm performance. The maximum candidacy and its CI for VT combinations with (+) and without (−) VT2 for each subject and all the subjects grouped together are represented. The maximum candidacy when all the input VTs are considered is shown in pink, as reference. The presence of VT2 returned similar results to the all VTs cases (pink data) for unevenness recognition ((**a**), purple data) and three-terrain identification ((**b**), blue data) in most of the cases.

The algorithm was evaluated with different combinations of the input VTs to investigate their contribution to human terrain choice. Table 3 and Table 4 report the maximum candidacy for each subject and input combination. The subject-wise candidacy and the corresponding candidacy CIs of the combinations with VT2 (+) and those without it (−) are shown in Figure 7. The information about the contribution of VT1 and VT3 is shown in Supplementary Materials Figures S3 and S4, respectively. For the unevenness recognition, the CI of the candidacy for all the subjects was [82.2%, 88.1%] when considering VT2 and [70.7%, 78.0%] without VT2. In the case of the terrain identification task, the CI of the candidacy was [56.6%, 64.8%] with VT2 and [46.8%, 55.1%] without VT2.

## 4. Discussion and Conclusions

This study presents a neuromorphic haptic feedback strategy for the recognition of uneven terrains. The tactile information of the foot–ground interaction during natural walking was captured and delivered to the user by means of VT units placed on the haptic belt worn on her/his waist. The progression of the gait was encoded in the spatial distribution of the foot pressure recorded by the sensing insole. Then, it was injected into slowly adaptive mechanoreceptor neuronal models and hence converted into spike trains that activated the spatially corresponding VT (see Section 2 and Figure 1d). The proposed neuronal strategy elicited a wave sensation on the waistline, travelling from the spine to the navel to trace the progressive phases of the stance (Figure 1a,b).

This study successfully demonstrates that the subjects rapidly learnt sensorimotor contingencies that govern terrain exploration through biomimetic neuromorphic haptic feedback. The duration of each walk was chosen to be close to the estimated duration of the haptic working memory [52], thus allowing the subjects to appreciate the relative similarities/differences in the VT patterns amongst the trials. The psychophysical recognition tests with this vibrotactile feedback substantiated the proposed contingency mimetics to provide embodied awareness of the terrain walked upon. These findings pave the way for new enabling strategies to be addressed for lower-limb amputees.

The vibrotactile haptic stimulations have been often exploited to non-invasively restore the missing plantar sensory information in amputees [15,19,35,36,53]. Partially fulfilling the laws of sensorimotor contingencies (SMC), the activations of the tactile neuronal pathway build their corresponding natural internal representation and provide the accountable sensory perception [40,52,54]. However, the extent of these natural tactile perceptions for lower-limb sensory augmentation has rarely been challenged. More specifically, the quality of the haptic information is biomimetically extended in the presented study, allowing the subjects to reliably discriminate between even and uneven terrains (accuracy_H_: 87.5% ± 11.7%). On the other hand, the precise terrain identification was not reliably achieved, given the high similarity between tiles and grass and the related elicited sensations and effects (the relevant stance durations did not reveal significant differences; accuracy_H_: 62.5% ± 15.7% with reference to Figure 4). In the perspective of enhancing these results, further investigations with more terrain textures (e.g., gravel, sand, etc.) will be considered in future experiments. In addition, a continuous use of feedback during daily activities might consolidate the training and achieve better recognition performance. Notwithstanding the reduced ability of the participants to identify very similar grounds, one of the major difficulties for lower-limb amputees using a prosthesis is to maintain a balanced gait on an uneven terrain due to the lack of sensory information from the foot–ground interaction [54,55]. The results of the presented perceptual recognition test showed that, for the first time, a vibrotactile sensory augmentation strategy could primarily enable unevenness recognition and additionally support terrain identification. These achievements might ultimately mitigate the risk of falling in lower-limb amputees and influence their walking behaviors depending on the stepped-on-ground features. Further human-in the-loop trials will be considered to investigate the effect of stimuli identification on cognitive load during walking on uneven surfaces. In this regard, previous results have already demonstrated that healthy subjects were able to accurately localize vibrations during constrained dynamic conditions, i.e., treadmill walking [48].

To better understand the human decoding mechanism of the biomimetic sensory feedback approach, an algorithm was trained to classify the proposed terrains. The deactivation of VT1 and the activation of VT2 and VT3 were chosen as input features since they encoded the information about the progression of the foot-flat phase, which is crucial to detect under-the-foot obstacles. These features were extracted at each value of temporal scale, as shown in the examples of Figure 3. The increase in the bin size, as a percentage of the stance cycle, meant that variations in each bin were averaged over the corresponding time interval. Consequently, the input features for the KNN algorithm were progressively associated with a greater loss of information, determining lower machine recognition accuracy for larger bins (Figure 5 and Figure 6). The algorithm and the human subjects performed similarly in the recognition of both the unevenness and the terrains (Figure 5 and Figure 6). Hence, it can be inferred that the machine successfully extracted the haptic perceptual complexity that was learnt by the human subjects during the augmentation trials. Assuming this, the temporal resolution (CTRp), i.e., the temporal bin size, was extracted where the candidacy was the highest, since it represented the maximum correspondence between the algorithm output and the subjects’ responses. It resulted in 45 ms for the unevenness recognition and 15 ms for the terrain identification. However, the accuracy_A_ and the candidacy intersected the subjects’ CIs up to a bin size of approximately 25%, i.e., a temporal scale of 220 ms, in both the cases (Figure 5). Taking this into account, we can reasonably state that this value can be considered the upper bound of the human vibrotactile stimuli temporal resolution. When dealing with the subject-wise results, the CTRc followed the expected behavior, being greater for the unevenness recognition, which requires less-precise feature decoding. Nevertheless, we observed the absence of a significant slope in the relation between candidacy and the bin size for all the subjects except for subject 4 (Figure 6). Up to the maximum explored bin size, the algorithm accuracy_A_ and the candidacy remained in the range of most of the subjects’ CIs. Therefore, we conclude that the proposed method does not allow us to draw strong conclusions about the individual temporal resolutions (CTRc).

The influence of each VT unit on the human terrain recognition choice was also investigated. It was observed that the information provided by VT2, whose activation occurred during the foot-flat event, was crucial for the algorithm to match the human responses. Differently from the VT subsets that included VT1 and VT3, those with VT2 matched the candidacy values achieved when using all the available inputs and were considerably better than the subsets without it (unevenness recognition: (+VT2) CI = [82.2, 88.1]%, (−VT2) CI = [70.7, 78]%; terrain recognition: (+VT2) CI = [56.6, 64.8]%, (−VT2) CI = [46.8, 55.1]%; Figure 7). A similar pattern can be also observed when looking at individual subjects, except for subject 1 (Figure 7). For this subject, the most informative vibrotactile unit was VT1 (Supplementary Materials Figure S3). Despite the importance of the contribution of VT2, the other VTs carried useful information since, with reference to Table 3 and Table 4, the addition of VT1, VT3 or both led to a slightly greater match between the human and the algorithm responses. This can also be explained by the nature of the feedback itself, conceived to encode terrain features as well as stance phases.

Overall, this study demonstrated the possibility to identify the unevenness of the profile of the walked-upon terrains by means of a vibrotactile augmenting feedback device. The neuronal spiking model fostered the interpretation of the human decoding mechanism of the perceived stimuli. This outcome can further promote the refinement of the feedback patterns to enhance their effectiveness during explorative tasks. In this regard, lower-limb amputees will be recruited to experience the proposed approach and to validate the usability and the benefits of the biomimetic vibrotactile feedback system in rehabilitation and real-life scenarios.

## Figures and Tables

**Table 1:** Subject-wise algorithm performance for unevenness recognition.

		Accuracy_H_	Accuracy_A_	Candidacy
Subject 1	Even	90.6%	100%	93.5%
	Uneven	93.8%	75%	81.2%
	All	91.7%	91.5%	89.4%
Subject 2	Even	68.8%	93.8%	68.8%
	Uneven	75%	62.5%	87.5%
	All	70.8%	83.3%	75%
Subject 3	Even	93.8%	100%	93.8%
	Uneven	81.2%	68.8%	62.5%
	All	89.6%	89.6%	83.3%
Subject 4	Even	100%	100%	100%
	Uneven	93.8%	100%	93.8%
	All	97.9%	100%	97.9%
All	All	87.5%	91.1%	86.4%

**Table 2:** Subject-wise algorithm performance for terrain identification.

		Accuracy_H_	Accuracy_A_	Candidacy
Subject 1	Tiles	50.0%	60%	53.3%
	Grass	56.2%	68.8%	87.5%
	Stones	93.8%	68.8%	68.8%
	All	66.7%	66%	70.2%
Subject 2	Tiles	18.8%	56.2%	18.8%
	Grass	37.5%	12.5%	56.2%
	Stones	75%	68.8%	75%
	All	43.8%	45.8%	50%
Subject 3	Tiles	37.5%	31.2%	56.2%
	Grass	56.2%	43.8%	43.8%
	Stones	81.2%	81.2%	75.0%
	All	58.3%	52.1%	58.3%
Subject 4	Tiles	93.8%	87.5%	93.8%
	Grass	56.2%	75%	37.5%
	Stones	93.8%	100%	93.8%
	All	81.2%	87.5%	75%
All	All	62.5%	62.8%	63.4%

**Table 3:** Algorithm unevenness recognition performance for the different combinations of VTs. Maximum candidacy values and the corresponding accuracy_A_ in parentheses.

	Subject1	Subject2	Subject3	Subject4
VT1	91.5% (89.4%)	56.2% (68.8%)	68.8% (66.7%)	68.8% (66.7%)
VT2	83% (85.1%)	72.9% (85.4%)	83.3% (89.6%)	95.8% (97.9%)
VT3	87.2% (89.4%)	68.8% (77.1%)	70.8% (72.9%)	77.1% (79.2%)
VT1-VT2	91.5% (89.4%)	72.9% (85.4%)	83.3% (89.6%)	97.9% (100%)
VT1-VT3	87.2% (89.4%)	68.8% (77.1%)	72.9% (75%)	77.1% (79.2%)
VT2-VT3	87.2% (89.4%)	75% (83.3%)	83.3% (89.6%)	97.9% (100%)
All VTs	89.4% (91.5%)	75% (83.3%)	83.3% (89.6%)	97.9% (100%)

**Table 4:** Algorithm terrain identification performance for the different combinations of VTs. Maximum candidacy values and the corresponding accuracy_A_ in parentheses.

	Subject1	Subject2	Subject3	Subject4
VT1	57.4% (59.6%)	37.5% (52.1%)	37.5% (39.6%)	54.2% (60.4%)
VT2	48.9% (53.2%)	52.1% (52.1%)	54.2% (50%)	72.9% (87.5%)
VT3	59.6% (63.8%)	41.7% (52.1%)	39.6% (39.6%)	66.7% (68.8%)
VT1-VT2	70.2% (72.3%)	50% (60.4%)	56.2% (54.2%)	75% (89.6%)
VT1-VT3	59.6% (59.6%)	41.7% (47.9%)	50% (43.8%)	66.7% (72.9%)
VT2-VT3	57.4% (63.8%)	50% (60.4%)	66.7% (56.2%)	75% (87.5%)
All VTs	70.2% (66%)	50% (45.8%)	58.3% (52.1%)	75% (87.5%)

## Data Availability

The data presented in this study are available on request from the corresponding author. The data are not publicly available due to ethical restrictions.

## Supplementary Materials

The following supporting information can be downloaded at https://www.mdpi.com/article/10.3390/s23094521/s1. Figure S1: Analysis of the subjects’ stance length; Figure S2: Subject-wise psychophysical results; Figure S3: Effect of VT1 on algorithm performance; Figure S4: Effect of VT3 on algorithm performance.
