# Supplementary material for: Uneven Terrain Recognition Using Neuromorphic Haptic Feedback
Source: Sensors (Basel). 2023 May 6;23(9):4521. doi: 10.3390/s23094521 (PMC10181691; doi:10.3390/s23094521)
Supplement: Supplementary file 1 [file sensors-23-04521-s001.zip › sensors-2316385-supplementary.pdf]

Article

Uneven Terrain Recognition Using Neuromorphic Haptic Feedback

Sahana Prasanna [1,2,†], Jessica D'Abbraccio [1,2,†], Mariangela Filosa [1,2,3,*], Davide Ferraro [1,2], Ilaria Cesini [1,2], Giacomo Spigler [1,2], Andrea Aliperta [1,2], Filippo Dell'Agnello [1,2], Angelo Davalli [4], Emanuele Gruppioni [4], Simona Crea [1,2,3,5], Nicola Vitiello [1,2,3,5], Alberto Mazzoni [1,2] and Calogero Maria Oddo [1,2,3,*]

1 The BioRobotics Institute, Sant'Anna School of Advanced Studies, 56127 Pisa, Italy; prasanna.sahana.3@gmail.com (S.P.); dabbracciojessica@gmail.com (J.D.); ferraro.dav@gmail.com (D.F.); ilariacesini91@gmail.com (I.C.); spiglerg@gmail.com (G.S.); andrea.aliperta@gmail.com (A.A.); filippo.dellagnello@santannapisa.it (F.D.); simona.crea@santannapisa.it (S.C.); nicola.vitiello@santannapisa.it (N.V.); alberto.mazzoni@santannapisa.it (A.M.)

2 Department of Excellence in Robotics & AI, Sant'Anna School of Advanced Studies, 56127 Pisa, Italy

3 Interdisciplinary Research Center Health Science, Sant'Anna School of Advanced Studies, 56127 Pisa, Italy

4 Centro Protesi INAIL (Italian National Institute for Insurance against Accidents at Work), 40054 Budrio, Italy; a.davalli@inail.it (A.D.); e.gruppioni@inail.it (E.G.)

5 IRCCS Fondazione Don Carlo Gnocchi, 50143 Florence, Italy

* Correspondence: mariangela.filosa@santannapisa.it (M.F.); calogero.oddo@santannapisa.it (C.M.O.)

† These authors contributed equally to this work.

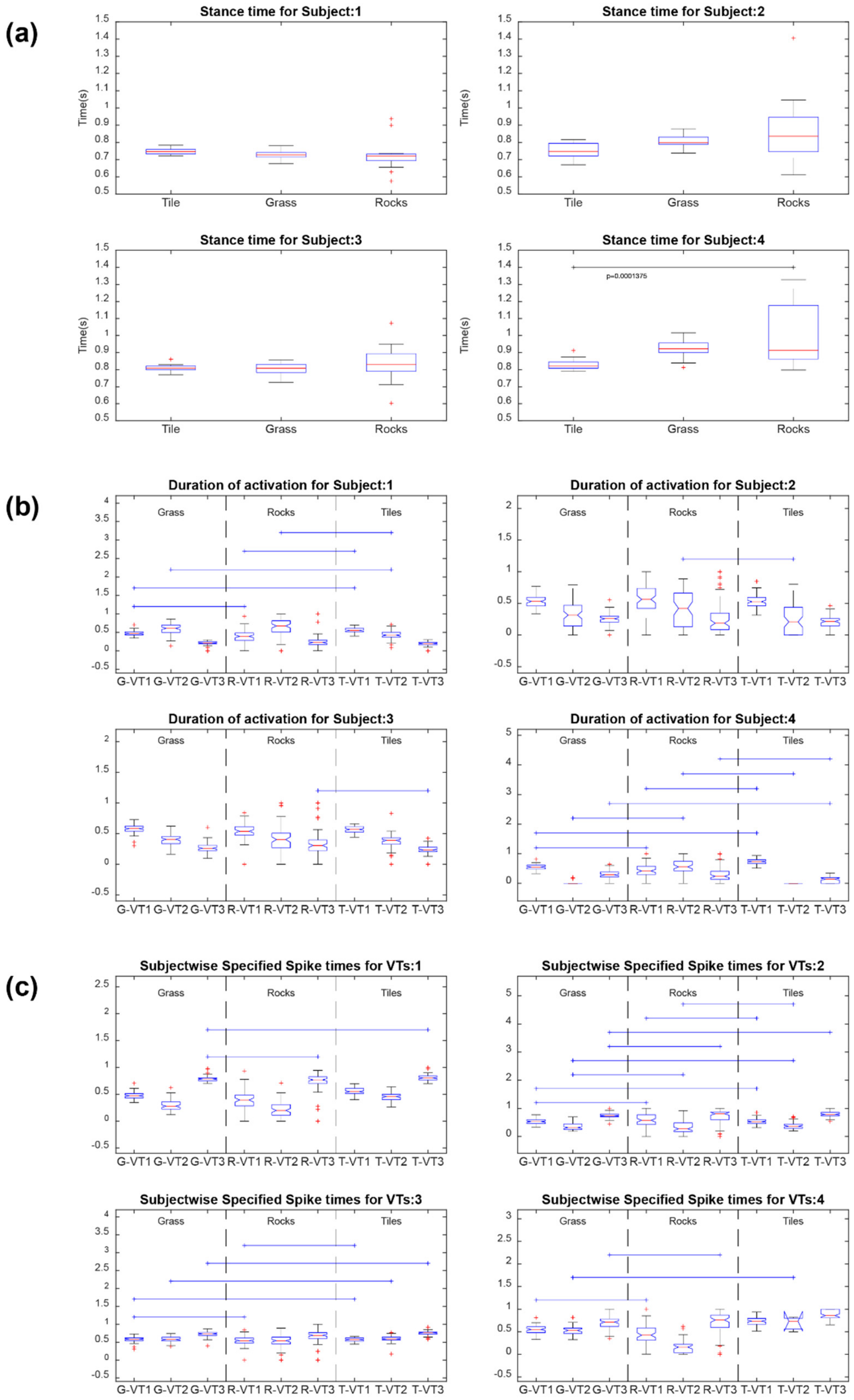

Figure S1. Analysis of the subjects' stance length. a) Subject-wise plot of the time taken to complete the stances with the significant difference (p≤0.05); (b) Results of the ANOVA test on the activation duration of the VT units. The significant differences (p≤0.05) between the corresponding VTs across the terrains are only displayed (c). The ANOVA results of the spike times corresponding to the last activation of VT1 and the first activations of VT2 and VT3, representing the lowest resolution of the bin size corresponding to 15ms for the PSTH.

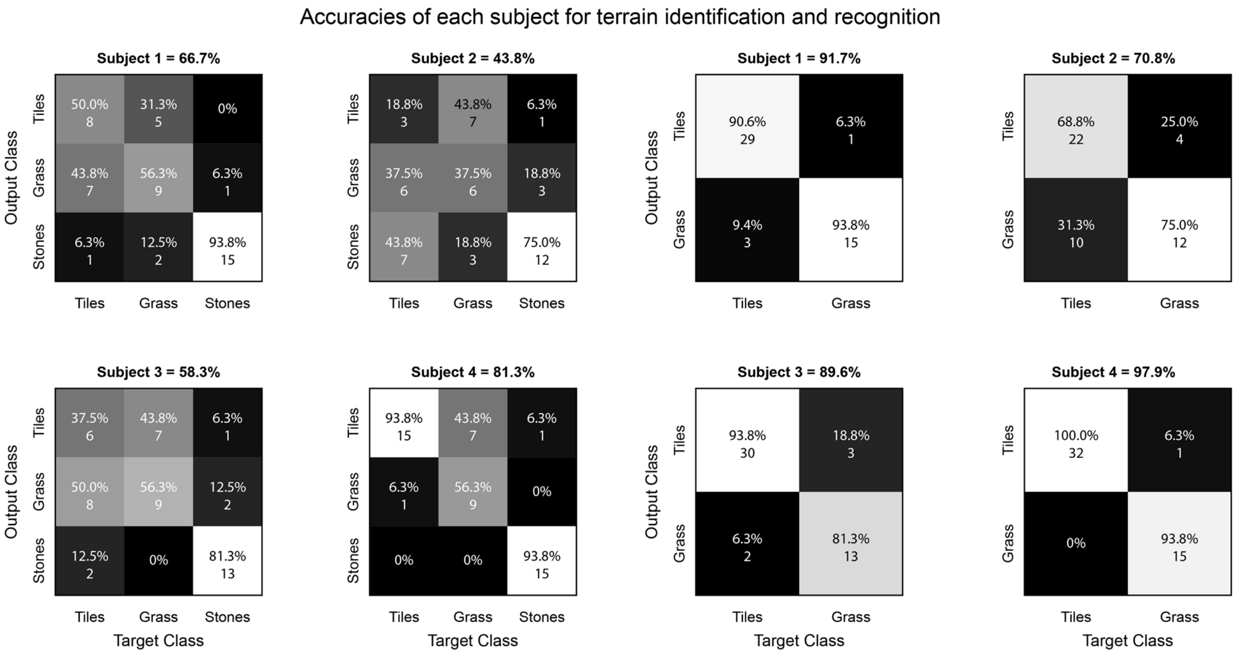

Figure S2. Subject-wise psychophysical results. The presented stimulus is denoted by target class and response by output class: (a) Confusion matrices of identification of tiles, grass or stones; (b) Confusion matrices of recognition of even terrain (tiles or grass) with respect to uneven (stones).

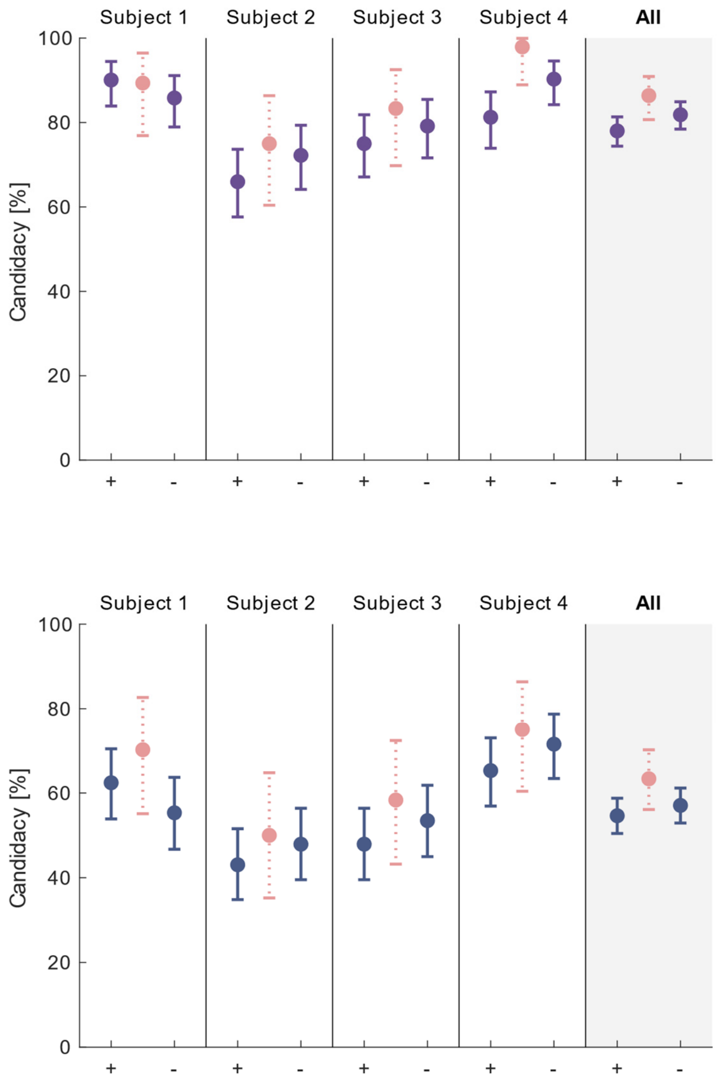

Figure S3. Effect of VT1 on algorithm performance. Algorithm maximum candidacy and CI for the VT combinations with (+) and without (-) VT1 for each subject and when considering all of them. The maximum candidacy when all the input VTs are considered is shown in pink as reference. Top: Unevenness recognition; Bottom: 3-terrain identification.

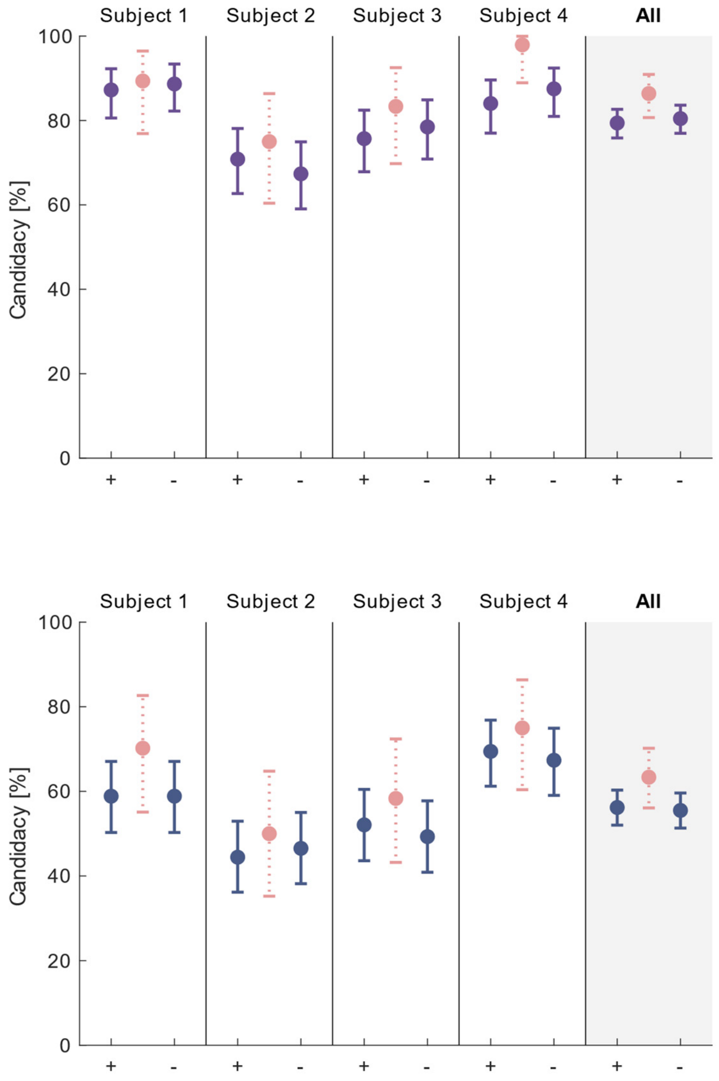

Figure S4. Effect of VT3 on algorithm performance. Algorithm maximum candidacy and CI for the VT combinations with (+) and without (-) VT3 for each subject and when considering all of them. The maximum candidacy when all the input VTs are considered is shown in pink as reference. Top: Unevenness recognition; Bottom: 3-terrain identification.
